# Sustainable Food Systems and Food Market

**DOI:** 10.3390/foods13131962

**Published:** 2024-06-21

**Authors:** Charles Odilichukwu R. Okpala

**Affiliations:** UGA Cooperative Extension, College of Agricultural and Environmental Sciences, University of Georgia, Athens, GA 30602, USA; charlesokpala@gmail.com

Within the realms of sustainability, it is well-established that food systems increasingly appear through the components of consumption, production, and processing of (food) products. In recent years, there has been a continued debate on sustainable food systems, which has helped to strengthen various governments’ pursuits to enhance the citizenry’s health and well-being around the globe [1]. Achieving a hunger-free world is core to the well-known Goal Two (2) (of seventeen) from the 2030 Agenda for Sustainable Development, which was adopted by all United Nations members in 2015 [2]. Broadly speaking, however, it is important to understand that attracting consumers to purchase any given food product should be dependent on such factors as affordability, availability, consumer need, convenience, logistics, (food) product type, quality, as well as quantity [3]. Another instance considers when consumers are making the decision to purchase a given food product, where they would think about the concept, content, and context perspectives of agrifood product quality [4]. In such a situation, the quality of two of the same agro-food products obtained from any given batch would most likely not be exactly the same. As part of the (food) supply chain, therefore, food markets occupy a critical position, especially in attracting consumers to a given food product [5].

From the local/national to international perspectives, how the global food system would operate remains defined by several characteristics, which are very important given their capacity to facilitate the (global food system’s) progress, from environmental concerns/issues, influential measures/scales, through the production cycles, social levels, and supply chains, to the various purchase locations/points [3,6]. The influence that these above-mentioned components have on the food market most likely determines the extent to which farmers can effectively and efficiently produce and supply their products to the consumer’s shelf. On this premise, this current Special Issue has facilitated some brainstorming on what brings sustainable food systems and food markets together, from the challenges that influence food product storage in food markets, to the extent to which food retail has been effectively positioned within the local/global sustainable (food) systems. A total of six publications are featured, bringing promising perspectives to this Special Issue, each of which is succinctly presented below.

Through the context of direct application for agri-food value chains, Constantin and colleagues proposed the construction of a sustainable economic competitiveness index (SECI), which looked at three attributes: agricultural chain performance, factor endowments/resource independence, and national agricultural chain strategies/policies. These workers tested the SECI against the cereal chain using selected EU countries helped by data from FAOSTAT/INTRACEN Trade Map between 2011 and 2020. The contributions of these authors stand as proof that building sustainable agricultural economic competitiveness relies on a mix of strategic actions. The key vector in this mix is that trade flow patterns and policies must be calibrated following national factor endowments to achieve SECI results that are at highly promising levels.

Damico and colleagues made an effort to deduce the attitude of Argentine zoomers alongside how they perceive sustainable food production. For clarity, zoomers were defined as consumers entering the market for the first time who were expressing their preferences without being guided by their parents. Two key aims guided the authors in this piece of research. Firstly, the extent of knowledge these new consumers have about sustainability to make choices that concern them. Secondly, whether they can push the market towards making a change. The consumers in this specific work were found to have high concern regarding the planet’s health (87.9%), followed by concern around the unsustainable methods of production (88.8%), followed by high interest associated with sustainable agriculture products, which quite a high percentage of consumers might be willing to pay for (74.1%).

Madeira and colleagues applied a time-out context to seek how fascinated tourists were with food markets specific to an urban area in Lisbon, Portugal. This involved a questionnaire evaluation of participants who had visited and experienced the market food court via perceptions of the locale’s cultural attractiveness, food quality/neophilia, location, as well as market engagement. The contributions of these authors were that food neophilia as depicted by visitors’ satisfaction showed the highest impact, which not only moderated their relationship of satisfaction (with market revisit) but positively influenced their intent to revisit the market. In addition to tourist attractions, modern food markets make an important contribution to the economy, especially via urban city regeneration.

Food markets would have one form of vegetable or another for sale, and evaluating its consumer safety is crucial to sustainable food systems [7]. To estimate the associated health risks that would arise for local consumers, Pan and Han investigated cadmium (Cd) concentrations in over 2000 vegetable samples gathered from over 50 species between 2018 and 2022. The contributions of these authors showed that, despite Cd levels significantly varying across sampling areas and years, there appeared an average Cd concentration of 0.035 mg kg^−1^ with bulb vegetables having higher relative accumulation compared to others. These workers deemed Cd levels to be of low health risk through vegetable consumption and reiterated the need for routine monitoring (of Cd levels) for consumer food safety/public health.

As mentioned earlier, achieving a hunger-free world is core to the well-known Goal Two (of seventeen) from the 2030 Agenda for Sustainable Development ([2]). It is towards this goal that Silva, Pedrozo, and Silva articulated a Sustainable Food System situation in Brazil, which involved the Brazilian National School Feeding Program (Programa Nacional de Alimentação Escolar—PNAE). These workers employed a framework approach to analyze the lessons learned from PNAE specific to its contributions to the sustainable food system’s development in Rio Grande do Sul (Brazil). The framework approach involved a qualitative descriptive survey where abductive logic allowed for multiple cases and semi-structured interviews. The contributions of these authors revealed how individuals could be grouped by learning level(s), strictly following the rules as prevailed by instrumental learning. Groups with communicative learning seemed more proactive and would improve.

Considering agri-food products within a sustainable supply chain, Wang and colleagues examined influencing factors based on cross-border live-streaming e-commerce in China. These workers considered three aspects in their work: (a) live streaming cross-border to delineate essence/fundamental characteristics of sustainable agricultural product supply chains; (b) the combination of platform and ecosystem theories from a grounded context as a primary research method; as well as (c) developing sustainable cross-border agricultural product supply chain pathways using live-streaming e-commerce that provides relevant stakeholders with required decision-making support from government agencies and practitioners perspectives. The contributions of these authors were on how the expansion of live-streaming e-commerce and sustainable cross-border ecosystems within agri-product supply chains would provide theoretical support for cross-border establishments/operations.

Whereas the components of the global food market remain very interconnected by function and structure, there would still be a number of meeting points where the different domestic and global drivers (of food market) converge [8,9]. Overall, this current Special Issue has shown that, for sustainable food systems to thrive across various communities around the globe, the food market remains crucially yet strategically positioned as a developmental catalyst. Through the qualitative, quantitative, and mixed methods applied across the diverse pieces of scholarly research reported in this Special Issue, consumers can be said to be a progressive force embedded in the food markets’ produce, production, processing, and retail/wholesale processes, as well as its quality standards.
